# Physicochemical and biological characterization of 1E10 Anti-Idiotype vaccine

**DOI:** 10.1186/1472-6750-11-112

**Published:** 2011-11-22

**Authors:** Yoan J Machado, Yamilet Rabasa, Raquel Montesinos, José Cremata, Vladimir Besada, Dasha Fuentes, Adolfo Castillo, Kathya R de la Luz, Ana M Vázquez, Martin Himly

**Affiliations:** 1 Center for Molecular Immunology, 216 St and 15th Ave., Atabey Siboney, Playa, P.O. Box 16040, Havana 11600, Cuba; 2 Center for Genetic Engineering and Biotechnology, 31st Ave and 158 St, Playa, P.O. Box 6162, Havana 10600. Cuba; 3 Centro Nacional Para la producción de Animales de Laboratorio, La Habana, Cuba; 4 Christian Doppler Laboratory for Allergy Diagnosis and Therapy, University of Salzburg, Austria

## Abstract

**Background:**

1E10 monoclonal antibody is a murine anti-idiotypic antibody that mimics N-glycolyl-GM3 gangliosides. This antibody has been tested as an anti-idiotypic cancer vaccine, adjuvated in Al(OH)_3_, in several clinical trials for melanoma, breast, and lung cancer. During early clinical development this mAb was obtained *in vivo *from mice ascites fluid. Currently, the production process of 1E10 is being transferred from the *in vivo *to a bioreactor-based method.

**Results:**

Here, we present a comprehensive molecular and immunological characterization of 1E10 produced by the two different production processes in order to determine the impact of the manufacturing process in vaccine performance. We observed differences in glycosylation pattern, charge heterogeneity and structural stability between *in vivo*-produced 1E10 and bioreactor-obtained 1E10. Interestingly, these modifications had no significant impact on the immune responses elicited in two different animal models.

**Conclusions:**

Changes in 1E10 primary structure like glycosylation; asparagine deamidation and oxidation affected 1E10 structural stability but did not affect the immune response elicited in mice and chickens when compared to 1E10 produced in mice.

## Background

Anti-idiotype vaccination represents an innovative approach to target tumor-associated antigen-expressing cells. This approach comes directly from Jerne's idiotypic network theory, which postulates that due to the huge potentiality for diversity of the immunoglobulin variable regions, the idiotype repertoire can mimic the universe of self and foreign epitopes [1].

NeuGc-containing gangliosides are attractive targets for cancer immunotherapy because these glycolipids are non-self antigens in humans [2,3]. In contrast, they have been detected in different human tumors by antibodies and chemical analysis [4-6]. Recent experimental data suggest that N-glycolyl-GM3 ganglioside (NeuGcGM3) is relevant for tumor biology [7].

mAb-1E10 [8] is an IgG1 anti-idiotype (Ab2) mAb obtained by immunizing Balb/c mice with mAb-P3 (Ab1) [9] coupled to keyhole limpet hemocyanin (KLH) in the presence of Freund's adjuvant. This Ab2 inhibited the binding of mAb-P3 to NeuGcGM3 ganglioside. mAb-1E10 induced an idiotype-positive antigen-negative (Id+Ag-) Ab3 response in syngeneic, allogeneic and xenogeneic models, where NeuGc-containing gangliosides are normally expressed [8,10]. In contrast, in chicken, where like in humans NeuGc-containing gangliosides are not expressed in normal tissues, mAb-1E10 was capable of inducing a specific Ab3 antibody response against these gangliosides (Id+Ag+) [10]. Similar results have been obtained in cancer patients immunized with Al(OH)_3_-precipitated mAb-1E10 [11-14]. The results of these clinical trials evidenced that the vaccine was well-tolerated and immunologically active. In addition, Al(OH)_3_-precipitated mAb-1E10 immunization induced a pronounced anti-metastatic effect in different murine tumor models [15,16].

For phase I and II clinical trials, mAb-1E10 was produced in mice ascites, a common practice in the 1990's for small scale antibody production.

We developed a new bioreactor-based method using protein-free media for the production of mAb-1E10. The mAb-1E10 produced from bioreactors (1E10-ST) has to be bioequivalent to ascites fluid-produced 1E10 (1E10-AF) in order to ensure the same effect in the patients. In this case, this bioequivalence has to be demonstrated by a set of physicochemical and biological methods as required by regulatory authorities for characterization of mAbs [17]. As mAb-1E10 is used as an adjuvated vaccine additional characteristics should be taken in to account.

Defining the molecular similarity of two mAbs can be difficult due to their inherent heterogeneity. Apart from the primary sequence, it has been established that glycosylation can be critical for the biological function of mAbs [18-21]. Product-related substances or impurities such as deamidated, isomerized, and oxidized forms, or protein aggregates [22-25] that may be introduced during cloning and production processes can affect, both, the mAbs' tertiary structure and antigen-binding properties. Therefore, a detailed characterization has special relevance for idiotypic vaccines, where the correct spatial atomic distribution in the Complementarity-Determining Regions (CDRs) is critical for their biological activity.

Here, we present the detailed molecular and immunological characterization of mAb-1E10 obtained by two different production methods in order to determine the impact of the manufacturing process in vaccine performance.

## Results and Discussion

### N-terminal pyroglutamic acid, Asn glycosylation and three deamidation sites common for 1E10-AF and ST, while oxidized methionine found only in 1E10-ST

**Figure 1:**
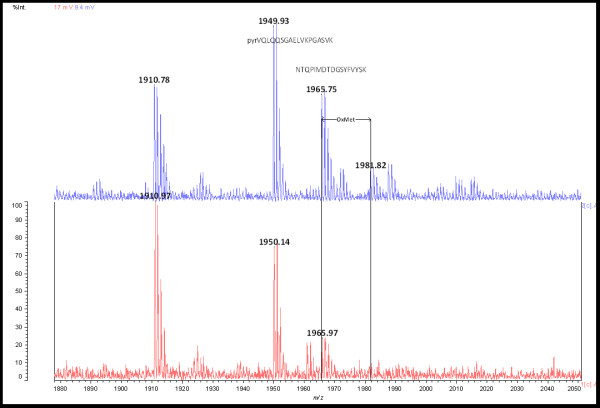
**Representative MALDI-TOF spectrum of 1E10 ST (upper panel) and 1E10 AF (lower panel)**. Sequences containing N-terminal pyroglutamic acid and methionine 396 are shown at their corresponding m/z. The 16 Da mass shifts corresponding to methionine oxidation is illustrated. Mass values corresponding to methionine oxidation were not detected in 1E10 LAM.

Primary structure was determined by mass spectrometry using both MALDI-TOF^2 ^and ESI-QTOF for MS^2 ^measurements. The 1E10 amino acid sequence remained unaltered during stirred tank fermentation or production in ascites fluid. Post-translational modifications detected were heavy chain N-terminal pyroglutamic acid, N-glycosylation and the oxidation of methionine 396 (Figure 1), as summarized in Table 1.

**Table 1:** PTMs of mAb-1E10 detected by mass spectrometry.

Modifications	1E10 AF	1E10 ST
N-term glutamine to pyroglutamic acid	+	+

Glycosylation of Asn294	+	+

Deamidation of Asn141 (heavy chain)	+	+

Deamidation of Asn157 (light chain)	+	+

Deamidation of Asn161 (light chain)	+	+

Oxidation of M396	+	-

+ Modified.-Non Modified

**Figure 2:**
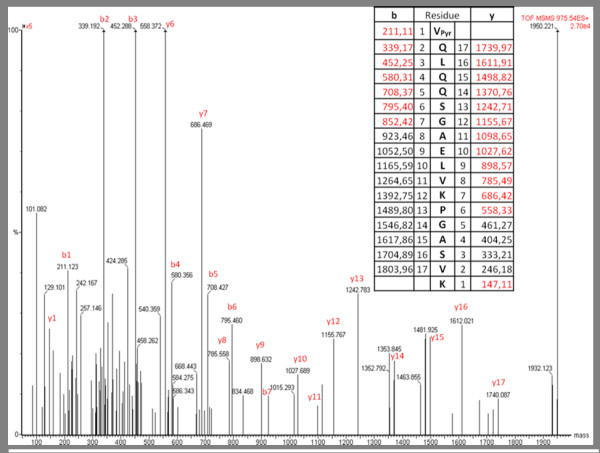
**Deconvoluted ms/ms spectrum of heavy chain N-terminal peptide containing pyroglutamic acid**. y and b fragment ions assigned are shown in the table and spectrum.

One common post-translational modification observed in mAbs is a cyclized N-terminal glutamine [24,26-28] when either the heavy and/or light chain sequences begin with glutamine. This reaction involves the cyclization of the N-terminal amine and the subsequent loss of NH_3 _(17 Da). The gene sequence of the heavy and light chains of many mAbs codes for an N-terminal glutamine, but upon sequencing the N-terminal amino acid is predominantly found as the cyclized pyro-glutamic acid form [24,27,29]. Figure 2 shows the MS^2 ^mass spectrum of the N-terminal peptide from heavy chain which bears the cyclized pyro-glutamic acid form of glutamine. Regardless the source, ascites or stirred tank, all the heavy chain N-terminal glutamines were found as pyro-glutamic acid, exclusively.

Methionine oxidation is a chemical modification known to change the stability and conformation of mAbs through altering CH2 and CH3 domain structure [30,31]. Figure 1 shows the oxidation in residue Met_396_, which is a conserved residue located at CH3 of IgG1 antibodies, near to the region of interaction between CH2 and CH3 domains. This oxidation affects the overall charge and stability of the antibody [32].

Deamidation has been shown to be one of the major chemical degradation mechanisms in protein pharmaceuticals during production and storage [33] Three sites of Asn deamidation were found for mAb-1E10, two of these were found in the constant region of the light chain and the other one in the constant region of heavy chain, two of these sites were also observed by Harris et al. [25] studying MMA383 anti-idiotypic antibody.

### Very low amounts of aggregates in 1E10-AF and ST, but low molecular weight fragments present only in 1E10-AF

**Figure 3:**
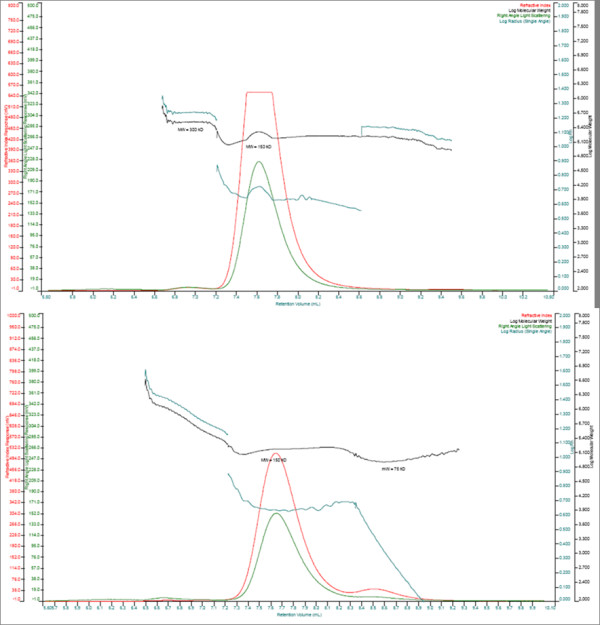
**Aggregation analysis of 1E10. HPSEC-TDA chromatograms of 1E10 ST (upper) and 1E10 AF (bottom)**. Refractive index (red), Log Molecular Weight (black), Right Angle Light Scattering (green) and Log Radius (pale green) signals are shown. 1E10 ST revealed one major peak corresponding to > 98% of monomers and a small amount of dimmers (< 2%). Two major peaks corresponding to > 91% monomeric 1E10 eluting and 7.9% of fragments with masses around 75 kDa were determined for 1E10 AF. Dimmers seemed to be < 1% for this mAb.

Aggregation analysis (Figure 3) revealed one major peak corresponding to > 98% monomeric 1E10-ST eluting at a retention volume of 7.6 mL. Additionally, a small amount of dimers (< 2%) seemed to be present (v_ret _= 6.9 mL). 1E10-AF showed two major peaks corresponding to > 91% monomeric 1E10 eluting at a v_ret _of 7.7 mL and 7.9% of mAb fragments (v_ret _= 8.6 ml) were determined. Dimers seemed to be < 1% (v_ret _= 6.9 ml).

Aggregates have been reported to cause immunogenicity in a T cell-independent pathway [34]. As aggregates comprise a large number of individual protein molecules, there is the potential for the surface to display a "repetitive" array of epitopes that may be seen by the immune system as resembling the external surfaces of pathogens, triggering the pattern recognition receptors (Toll-like receptors) on APCs. This T-cell-independent pathway leads to an IgM response. However, B cells activated by protein aggregates could function as APCs to recruit T cell help and thereby switch to an IgG response. Although B cells are relatively poor APCs, their presentation potential is enhanced following specific recognition of antigen, thus contributing to initiation of T cell responses [35].

Among the various product-related impurities and degradation products, aggregation is considered a strong risk factor for immunogenicity. It is also the attribute most difficult to control, because there are multiple factors that can lead to the formation of aggregates in a product [36].

### Charge profile of 1E10-ST different from 1E10-AF even after C-terminal lysine removal

**Figure 4:**
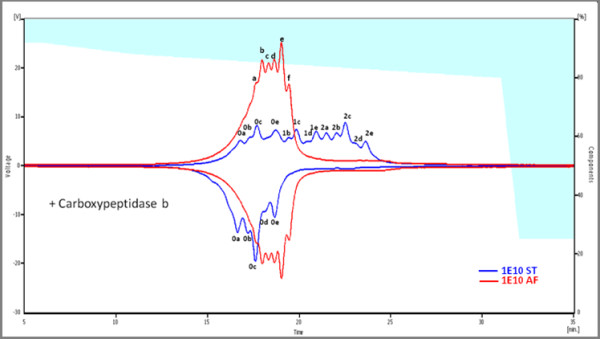
**WCX chromatograms of 1E10-ST (blue) and 1E10-AF (red)**. Gradient is represented in white (% A) and blue (% B), units are in the right axis. Inverted chromatograms were recorded after carboxypeptidase b treatment. Charged species are denoted with letters; prefix numbers indicate C-terminal lysine content for each peak. 1E10-ST gave rise to a continuous population of peaks due to the presence of terminal lysine while 1E10-AF showed almost no C-terminal lysine.

Cation-exchange chromatography has been introduced to measure charge heterogeneity, in particular caused by lysine variants, more than 15 years ago [37] and now is widely accepted in pharmaceutical analysis. We detected a complex charge variety for both molecules consisting of clusters of charged species (Figure 4). 1E10-AF remained unaltered after enzymatic removal of C-terminal lysine while in 1E10-ST the charged species changed dramatically. The shape of the WCX chromatograms and the changes observed after enzymatic treatment for both 1E10-AF and 1E10-ST suggested a complete processing of C-terminal lysine in 1E10-AF, while the 1E10 obtained from the stirred tank process was found to yield three charged populations of C-terminal lysine processing denoted in Figure 4 as 0 (mAb-1E10 with no C-terminal lysine), 1 (mAb-1E10 with C-terminal lysine partially removed), and 2 (intact mAb-1E10). Charged species present on each cluster (a to e in Figure 4) can result from posttranslational modifications like asparagine deamidation, N-teminal pyroglutamic acid and methionine oxidation determined by mass spectrometry. Harris et al [25] reported similar findings as potential sources for the significant charge heterogeneities in mAb-MMA383, an anti-idiotypic murine IgG1 mAb. In that study C-terminal lysine variants, proteolysis, and deamidation of light chain Asn_161 _and heavy chain Asn_141 _were identified to be responsible for mAb-MMA383 charge heterogeneity.

### Same sugar structures attached to Fc of 1E10-AF and ST but not their relative amount

Detailed N-glycosylation was investigated by N-glycan mapping of fluorescence-labeled oligosaccharides released from the mAbs. The 2-AB-labeled glycans, released from mAbs by PNGase F, were separated using hydrophilic interaction chromatography (HILIC) with fluorescence detection for quantification. The N-glycans identified were core-fucosylated biantennary-complex-type oligosaccharides. Using GU values [38], the most intense peaks were identified as agalactosylated core-fucosylated biantennary (G0F) and monogalactosylated core-fucosylated biantenary (G1F) structures, respectively. Minor peaks with higher retention times were determined to be core-fucosylated mono- and di-sialylated biantennary structures, respectively.

**Figure 5:**
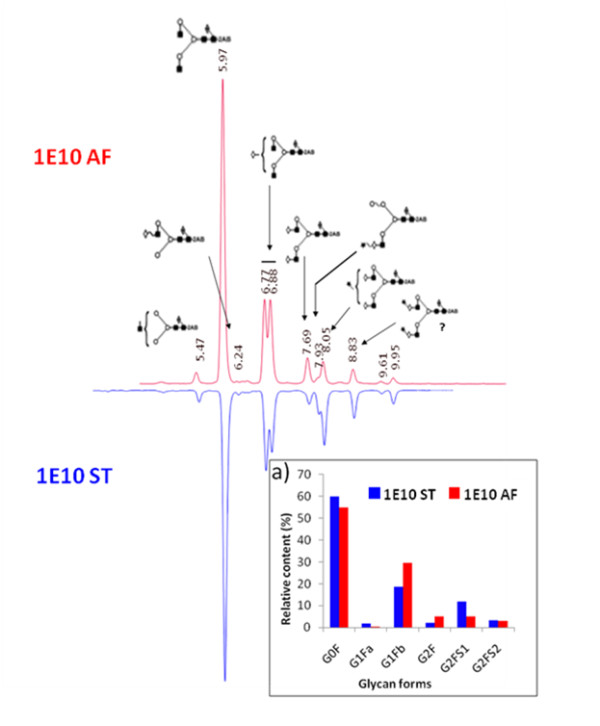
**Comparison of HPLC separation and quantification of 2-AB-labeled glycans released from 1E10 ST and AF**. a) Relative contents of 2-AB-labeled glycans quantified by peak integration of HPLC-fluorescence signals.

Oligosaccharide structures were determined to be similar for both mAb molecules. Six major glycans (G0F, G1Fa, G1Fb, G2F, G2FS1 and G2FS2) were identified and quantified. Figure 5a shows a relative quantification based on relative peak areas of 2AB-labeled HPLC chromatograms. As expected, G0F, G1F and G2 were the most highly represented glycoforms in both mAb-1E10, while sialylated glycoforms were less. Glycosylation patterns of 1E10-ST and AF were determined to be different in their ratios of glycan structures. However, they exhibited the same glycan structures attached to Asn_297 _of heavy chain. Compared to 1E10-AF, bioreactor-produced mAb contained more G0 and displayed a two-fold increase in sialylated forms. The glycan pattern of 1E10-AF can be a result from enzymatic trimming of oligosaccharides by glycosidases present in ascites fluid. However, the 1E10-ST glycan pattern may have resulted from differences in specific production rates of hybridoma cells during fermentation. At high production rates, mAb oligosaccharides result incomplete, while at low production rates, more complete structures are produced.

IgG glycosylation plays a crucial role in antibody effector functions like cell-dependent cytotoxicity (CDC), antibody-dependent cell cytotoxicity (ADCC), and FcγR recognition. As the intended use of mAb-1E10 is to be administered as a vaccine, adsorbed to alum, and injected subcutaneously, it is very unlikely that Fc-mediated effector functions plays a critical role in the biological activity of this mAb. However, conformation of IgG is affected by glycosylation [32]. On the other hand, it is well known that murine cells can attach highly immunogenic sugar epitopes like αGal-Gal and N-glycolyl sialic acid [21]. As the N-glycan structures of 1E10-AF and ST were identical, and so far none of these epitopes has been detected, differences in immunogenicity and safety between 1E10-AF and ST are not expected.

### Reduced thermal stability of 1E10-ST as compared to 1E10-AF

**Figure 6:**
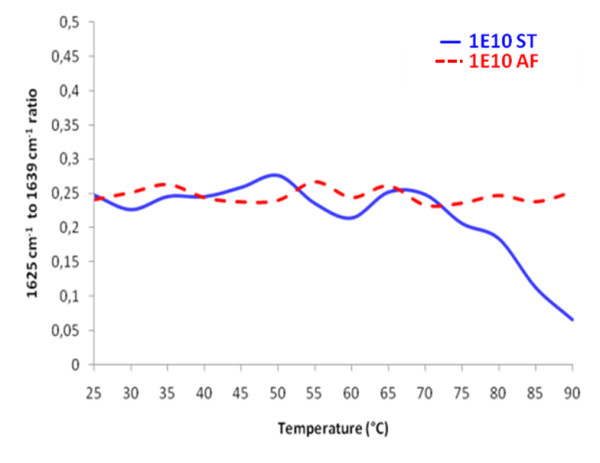
**Thermal transition curves of the 1E10-AF (dotted line) and 1E10-ST (solid line)**. Curves were obtained by plotting the ratio of the intensities of the increasing to the decreasing amide I band vs. temperature. 1E10-ST showed different thermal stability in respect to 1E10-AF.

Thermally induced structural transitions in the secondary structure monitored by infrared spectroscopy have been previously described [39-42] to analyze the unfolding behavior of proteins and to determine a melting temperature (Tm_FTIR_). As structural transition to intermolecular β-sheets occur regardless of the initial secondary structure composition of the native protein, these bands can be used to monitor protein aggregation in aqueous solution and solid state [33]. The melting point can be determined by plotting the ratio of the intensities of characteristic amide I bands vs. temperature [43]. 1E10-AF and 1E10-ST displayed a different behavior under temperature stress (Figure 6). While 1E10-AF remained in solution, 1E10-ST started melting at approximately 73°C. One explanation for the lower thermal stability of 1E10-ST might be the oxidized Met_396 _in 1E10-ST, which was not detectable in 1E10-AF. This residue is located on CH3 domain, near to the interaction site of CH2 and CH3, therefore, it may be hypothesized that oxidation of this residue can affect the three dimensional structures of CH3 and CH2 and herewith the overall stability of the antibody. In summary, the thermal stability for mAb-1E10 obtained in stirred tank fermentation was lower than mAb-1E10 obtained in ascites fluid, supported by several factors including methionine oxidation, differences in glycan patterns, charge state heterogeneities, and protein conformation.

### Similar Ab3 antibody responses elicited by 1E10-AF and ST vaccination in chicken

**Figure 7:**
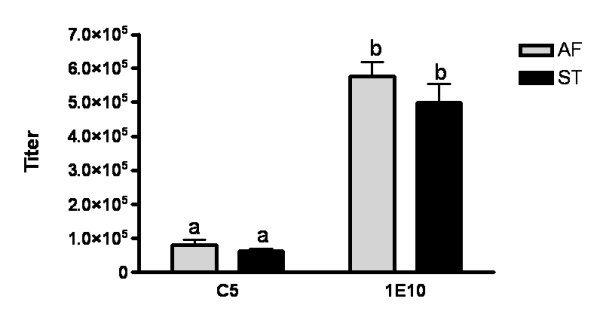
**Specificity of Ab3 antibodies to 1E10-AF and 1E10-ST in sera from immunized chicken determined by ELISA**. Chicken sera at different dilutions were bound to microtiter plates coated with 1E10-AF, 1E10-ST or isotype-matched control ior C5 mAb (10 μg/mL) and the reaction was developed with alkaline phosphatase-conjugated rabbit anti-chicken IgY. Sera were obtained seven days after animals received the third dose of each 1E10 mAb preparation. The data represent mean ± SD of maximal titers in chicken sera of each group.(p < 0:001, one way analysis of variance and Bonferroni multiple comparisons test). Similar letters means no difference (p > 0.05). Baseline serological antibodies were undetectable (< 100 titer).

We compared the specificity of the antibodies generated in chicken by immunization with 1E10-AF and ST. Antibody responses were tested in the sera obtained from the animals after the immunization. All chicken developed a strong humoral response against the whole mAb-1E10 molecules. The titers of Ab3 responses were higher than 1/400,000, as measured by ELISA (Figure 7). The reactivity of animal sera with the murine isotype-matched mAb was also tested, and a higher binding to the immunizing mAb-1E10 was observed in the chicken sera independently of the source of anti-idiotype mAb (Figure 7) (p < 0:001, one way analysis of variance and Bonferroni multiple comparisons tests). This result demonstrated the immunodominance of the idiotype of both mAb-1E10 products in the antibody response induced in immunized chicken. Moreover, there was no difference in the serological antibody titers obtained when the animals were immunized with 1E10-AF or 1E10-ST (p > 0.05) (Figure 7), evidencing the same immunogenicity for these two mAb-1E10 molecules. Thus, the structural changes observed did neither affect overall immunogenicity nor the idiotypic immunodominance reported for this mAb [10].

### Co-administration of 1E10-AF/Alum or 1E10 ST/Alum with low-dose Cyclophosphamide induced anti-tumor effects in a mammary carcinoma model

**Figure 8:**
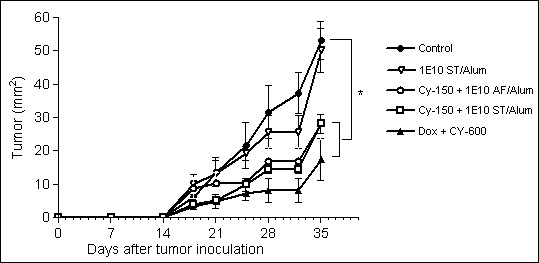
**Antitumor effects of 1E10-AF/Alum and 1E10-ST/Alum co-administrated with low-dose Cyclophosphamide in a mammary carcinoma model**. F3II tumor-bearing mice were immunized subcutaneously with 100 μg of 1E10-ST/Alum alone (open triangle); 1E10-AF/Alum (open circle) or 1E10-ST/Alum (open square) combined with 150 mg/m2 of Cyclophosphamide i.v. 7 days after the tumor inoculation. Both combinations reduced significantly the F3II mammary carcinoma growth. This response was comparable with the administration of high-dose chemotherapy based on 60 mg/m2 of Doxorubicin and 600 mg/m2 of Cyclophosphamide (full triangle). * Significant differences (P < 0.05) using Kruskal-Wallis and Dunn's multiple comparison tests.

As shown in Figure 8, no antitumor effect was observed when low-dose Cyclophosphamide or 1E10-ST/Alum was administered alone at the evaluated dose. Interestingly, when 1E10-AF/Alum or 1E10-ST/Alum was co-administered with low-dose Cyclophosphamide F3II mammary carcinoma growth was reduced significantly. No statistical differences in tumor growth between these groups of mice were determined and the observed anti-tumor responses were comparable with the effect upon co-administration of the standard high-dose chemotherapy for breast cancer based on 60 mg/m2 of Doxorubicin and 600 mg/m^2 ^of Cyclophosphamide.

*In vivo *experiments resulted in similar immunogenicity, idiotypic dominance, and antitumor effects of 1E10-AF and 1E10-ST. Thus, the observed posttranslational modifications did most likely not affect the Fab structure, as the biological activity of 1E10 vaccine was not altered. Moreover, deamidation of asparagine and methionine oxidation were restricted to Fc regions in 1E10 mAb, far away from CDRs. Therefore, it remains unlikely that these amino acid modifications might affect the spatial conformation of the respective CDRs enabling structural mimicry as postulated for anti-idiotypic mAbs.

## Conclusions

Effector functions of classic therapeutic monoclonal antibodies can be divided in Fab-dependent functions and Fc-dependent functions. The Fab region contains variable regions of light and heavy chains which are responsible for antigen recognition, while the Fc region mediates effector functions like CDC, ADCC and FcγR recognition. Therefore, any change in their physicochemical profile could eventually affect the efficacy of mAbs.

It is well known that physicochemical properties of recombinant proteins can be heavily affected during changes in the production process. Thus, it is mandatory to carefully investigate the similarities and differences of therapeutic mAbs when the production process has been changed. Comparability must be demonstrated by an array of physicochemical and biological methods as required by the regulatory authorities.

Regulatory aspects regarding physicochemical characteristics of idiotypic monoclonal antibodies used for idiotypic vaccination bear some differences from classic therapeutic mAbs. For idiotypic mAbs, Fc-related functions are not critical for the intended biological effect as they are administered adsorbed to alum and via intra-dermis. Antigen mimicry is restricted to variable region CDRs which are located in Fab.

Here, we demonstrate that change of 1E10 production from ascites to bioreactor generates a molecule with different physicochemical properties. Changes were observed in the degree of asparagine deamidation, C-terminal lysine processing, methionine oxidation, and glycosylation pattern. All these modifications had an impact on the charge profile and conformation of the 1E10 molecule, as revealed by an altered thermal stability of 1E10-ST. However, these changes did not affect its biological activity. The idiotypic dominance was not affected, which represents the intended effect for an idiotypic vaccine. Thus, the transfer in the production process of 1E10 idiotypic mAb from ascites to bioreactor improved product safety without affecting its biological activity. Furthermore, our study demonstrated a number of physicochemical properties that do not affect the biological activity of 1E10 idiotypic vaccine.

## Methods

### 1E10-AF

Hybridoma cell line was cultured in DMEM/F-12 medium supplemented with 5% of fetal calf serum (Invitrogen, USA), 5 mM of glutamine (Invitrogen, USA), 2.546 g/L of HEPES (Sigma, USA), 2.18 g/L of sodium bicarbonate (Sigma, USA) and 1 mL/L of 2-mercaptoethanol (Sigma, USA). Mouse ascites fluid from BALB/c mice was sterilized by depth filtration and 1E10 mAb was purified using the following chromatographic sequence: ion exchange by DEAE Sepharose, affinity chromatography using Protein A Sepharose, and Sepharose G-25 gel filtration chromatography (all from GE Heathcare, USA).

### 1E10-ST

Supernatant of 1E10-ST was obtained from the stirred tank bioreactors hybridoma cell culture using a proprietary protein free medium in perfusion mode. Clarified 1E10 supernatant was purified by protein A affinity chromatography using ProSep vA ultra matrix (Millipore, USA, followed by cation exchange chromatography (SP Sepharose FF, GE Healthcare, USA) and Sepharose G-25 size exclusion chromatography (GE Healthcare, USA).

### Aluminum hydroxide-precipitated 1E10-AF and 1E10-ST mAb formulations

Purified mAb-1E10 molecules were aseptically mixed at a final concentration of 1 mg/ml with 5 mg/ml of aluminum hydroxide as adjuvant (Superfos Biosector, Frederikssund, Denmark). The mixture was gently stirred for 3 h at room temperature. The aluminum hydroxide-precipitated mAb was stored in aliquots at 4°C until use.

### Amino acid sequence and posttranslational modifications (PTMs) analysis

Lyophilized mAbs (50 μg) obtained from each process were dissolved in 50 μL of 25 mM of Tris-HCl buffer, pH 8.5 with 8 M Urea. After addition of DTT to a final concentration of 50 mM, the mixture was flushed with N_2 _and incubated for 3 h at 37°C. Following this step the sample was cooled to room temperature, acrylamide was added to a final concentration of 100 mM and the mixture incubated for 20 min in the dark at room temperature. The samples were diluted 8 times to a final concentration of 1 M Urea. After addition of 1 μg trypsin, the mixture was incubated at 37°C for 18 h. The reaction was stopped by addition of 2% (v/v) TFA in water. The peptide mixture was analyzed directly by MALDI-MS and ESI-MS.

Analyses of peptides by MALDI-TOF^2 ^MS were performed on an Axima Performance mass spectrometer (Shimadzu Biotech, Japan) equipped with a 337 nitrogen laser and a collision-induced dissociation (CID) chamber with helium gas. Analyses were carried out in reflector mode (Mr < 5000) with delayed extraction. The instrument was calibrated externally using ProteoMass Peptide Calibration kit (Sigma), low mass gate value of 700 was selected. Data analyses were performed using Shimadzu Biotech Launchpad software (Shimadzu Biotech, Japan)

Digested peptides were purified using CleanUp Pippet Tips C18 (Agilent, USA) as recommended by the manufacturer. Sample was deposited on the MALDI plate by the dried-droplet sample preparation method [44] using α-cyano-4-hydroxycinnamic acid (CHCA), 10 μg uL^-1^, in TFA-water-acetonitrile (0.1:30:70, v/v).

ESI-MS and MS^2 ^were performed in the positive ion mode on a Micromass Q-TOF II mass spectrometer (Manchester, UK) equipped with a Z-spray sample introduction system. Acquisition and analysis of the data was performed using MassLynx software v 4.0 (Micromass, Manchester, UK). For calibration, a sodium and cesium iodide mixture solution was used.

### Aggregation analysis

High performance-size exclusion chromatography (HPSEC) of 100 μl sample was performed as standard using a 7.8 × 300 mm TSKgel G2000SWXL column protected by a 6 × 40 mm guard column (Tosoh Bioscience, Stuttgart, Germany) on a HP1100 analytical chromatography system (Hewlett-Packard, San Jose, CA, USA) at 0.5 ml/min in 100 mM sodium phosphate pH 6.6, 150 mM NaCl, 0.05% NaN_3_. Using a combination of the built-in UV detector measuring at 280 nm and a sequential refractive index (RI), intrinsic viscosity (IV), and right-angle light scattering detection (TDA 302, Viscotek Corp., Houston, TX, USA) the MW and hydrodynamic radius of eluting peaks were determined. Detector calibration was performed using bovine serum albumin from Sigma (A7638) weighed out at 1.0 mg/ml.

### Thermal stability of mAb-1E10 by FTIR

Infrared spectra were recorded using a Tensor 27 spectrometer (Bruker Optics, Ettlingen, Germany). Protein samples were prepared in a BioATR cell connected to a thermostat. For each spectrum, a 120 scan interferogram was collected in single beam mode at 4 cm^-1 ^resolution. Reference spectra were recorded under identical conditions with only the reference buffer in the cell. To determine the melting temperature (Tm_FTIR_), temperature-dependent spectra were acquired every 5°C in a ranging from 25 to 90°C. Recorded infrared spectra were analyzed by the Protein Dynamics software for Opus 4.2 (Bruker Optics) and displayed as vector-normalized second-derivative amide I spectra. Melting temperature was calculated by plotting the 1639 cm^-1 ^to 1625 cm^-1 ^signal ratio vs. temperature.

### Analysis of charge heterogeneity

1E10-ST and 1E10-AF (approximately 100 μg each) were diluted five-fold with buffer A (10 mM sodium acetate pH 5) loaded onto a ProPac10 WCX column (Dionex, Houston, USA) and eluted with buffer B (10 mM sodium acetate, 1 M NaCl pH 5). The gradient was performed in two steps, from 8 to 13% B in 5 min and 13 to 20% B in 20 min. Protein elution was monitored by absorbance at 280 nm.

The mAb solutions (1.5 mg/ml in 15 mM sodium phosphate pH 7) were treated with carboxypeptidase B (Boehringer, Germany) at an enzyme-to-substrate ratio of 1:5000 (w/w) at 25°C. Reaction was stopped by adding 1 μl of acetic acid. Digested samples were analyzed by WCX HPLC as described above.

### N-Glycosylation analysis

N-glycans were released by digestion with peptide-N4-(N-acetyl-β-d-glucosaminyl) asparagine amidase F (PNGase F, BioLabs, Beverly, MA, USA), using the method described by the manufacturer. Briefly, mAbs-1E10 were denatured at 100°C for 10 min in 0.1% SDS, 5% β-mercaptoethanol. Nonidet P-40 (NP-40) was added to a final concentration of 1% before enzyme addition. The digestion was carried out at a ratio of 5 U of PNGase F per milligram of glycoprotein at 37°C for 2 h. The protein was precipitated by adding three volumes of cold ethanol and the mixture was kept at -20°C for 30 min. The oligosaccharides were concentrated under vacuum and subjected to 2-aminobenzamide (2AB) labeling.

Oligosaccharides were fluorescently labeled with 2AB by reductive amination [45]. Briefly, oligosaccharides were dissolved in 5 μL DMSO-acetic acid (7:3) containing 2AB (0.35 M) and 1 M NaCNBH_3 _and incubated at 65°C for 2 h. Excess of fluorophore was removed by 2 h vertical chromatography on Whatman 3 MM paper using acetonitrile. The paper bearing the oligosaccharide signal (application point) was cut. The oligosaccharides were eluted from the paper by adding double-distilled water (2 × 500 μL). The eluate was filtrated in a syringe through a 0.45 μm PTFE filter (Millex-LCR, Millipore) and then concentrated under vacuum.

Normal-phase HPLC (NP-HPLC) of the labeled portion was performed using a TSK-Gel Amide-80 4.6 × 250 mm column (Tosoh BioSep, Japan) on a separation module (Merck-Hitachi, Japan) equipped with a fluorescence detector. Labeled N-glycans were separated by a linear gradient of 20-58% of 50 mM ammonium formiate pH 4.4 against acetonitrile over 152 min at a flow rate of 0.4 mL/min. Samples were injected in 80% acetonitrile. The fluorescence detection was carried out using an excitation wavelength of 330 nm and an emission wavelength of 420 nm [46]. The elution positions of the N-glycans were determined in glucose units (GU) by comparison with a standard dextran hydrolysate 2AB labeled (dextran ladder) [47].

### Animals

Female BALB/c/cenp mice (ten weeks old) and Leghorn chicken (10 weeks old) were purchased from the Center for Laboratory Animal Breeding (CENPALAB, Havana, Cuba). Animals were housed under conventional conditions with free access to water and food and maintained in accordance with the guidelines stipulated by the Animal Subject Committee Review Board of CENPALAB. Animal studies were performed with approval from CENPALAB's and CIM's Institutional Animal Care and Use Committees.

### Antitumor experiments

The sarcomatoid mammary carcinoma cell line F3II is a highly invasive and metastatic variant established from a clone of a spontaneous BALB/c mouse mammary tumor. F3II cells grow as spindle-cell carcinoma tumors with a high local invasiveness and a 90-100% incidence of lung metastases [48]. F3II cells were maintained in minimal essential medium (MEM 41500, Gibco, BRL) supplemented with 10% fetal bovine serum, 2 mM glutamine, 80 mg/ml Gentamycin, and 20 mg/ml tetracycline in monolayer culture.

All mice were injected subcutaneously with 2 × 10^5 ^F3II cells in the right flank. Seven days after tumor inoculation, the animals were separated in different groups and injected as follows: subcutaneously with normal saline; subcutaneously with 100 μg of 1E10-ST/Alum; intravenously with 150 mg/m^2 ^of Cyclophosphamide (Cy-150); intravenously with 150 mg/m^2 ^of Cyclophosphamide and subcutaneously with 100 μg of 1E10-AF/Alum; intravenously with 150 mg/m^2 ^of Cyclophosphamide and subcutaneously with 100 μg of 1E10-ST/Alum or intravenously with 60 mg/m^2 ^of Doxorubicin (Dox) and 600 mg/m^2 ^of Cyclophosphamide (Cy-600). The time of appearance of local tumors was monitored by palpation and further confirmed by histopathology. In all cases, tumors were diagnosed as spindle-cell carcinomas. Tumor size was measured with a caliper twice a week.

### Induction of anti-anti-idiotype antibody (Ab3) response

Chicken were immunized subcutaneously with 100 μg of 1E10 vaccine in the days 0, 7 and 21. Blood extractions were done before and one week after the last immunization (day 28).

The presence of anti-1E10 antibodies in chicken sera was determined in a solid-phase ELISA Polystyrene Maxisorp microtiter plates (Nunc, Roskilde, Denmark) were coated with 50 μl of a solution of 10 μg/ml of mAb-1E10 in carbonate buffer pH 9.6 and incubated overnight at 4°C. After washing with PBS containing 0.05% Tween 20, the plates were blocked for 1 h at room temperature with PBS containing 1% BSA. Then, diluted serum samples were added to each well and the plates were incubated for 2 h at 37°C. Chicken antibodies were detected with an AP-conjugated rabbit anti-chicken IgY (Sigma). The plates were washed four times and the reaction was developed with a substrate solution consisting of 1 mg/ml p-nitrophenylphosphate (Sigma) in diethanolamine buffer, pH 9.8. Absorbance was measured at 405 nm in an ELISA reader (Tekan, Salzburg, Austria).

The highest serum dilution giving OD values ≥0.2 and being at least three times the value corresponding to the preimmune serum at the same dilution was considered as titer. Assays were performed in triplicate for each sample and the coefficient of variation (CV) was less than 15%. The ODs of the blanks were less than 0.1.

## Authors' contributions

YJM conceived the experiments, carried out the MALDI-MS and charge heterogeneity determinations, the FTIR experiments, the immunogenicity studies, performed the overall data treatments and has drafted the manuscript. YR performed the ELISA determinations for all the immunogenicity studies. RM and JC performed glycans structure analysis; VB performed ESI-QTOF determinations and supported YJM in mass spectrometry data analysis. DF performed antitumor experiments and supported YJM in experimental design of animal experiments. AMV, AC, KRL and MH has been involved in the experiments planning, the drafting of the manuscript and for revising it critically for content and general supervision of the project. MH performed HPSEC-TDA determinations. All the authors have read and approved the final manuscript.
